# From Molecular to Cluster Properties: Rotational Spectroscopy of 2-Aminopyridine and of Its Biomimetic Cluster with Water

**DOI:** 10.3390/molecules26226870

**Published:** 2021-11-15

**Authors:** Adam Kraśnicki, Zbigniew Kisiel, Jean-Claude Guillemin

**Affiliations:** 1 Institute of Physics, Polish Academy of Sciences, Al. Lotników 32/46, 02-668 Warszawa, Poland; adamkrasnicki@yahoo.com; 2 Univ. Rennes, Ecole Nationale Supérieure de Chimie de Rennes, CNRS, ISCR-UMR6226, F-35000 Rennes, France; jean-claude.guillemin@ensc-rennes.fr

**Keywords:** hydrogen bonding, weakly bound clusters, rotational spectroscopy, supersonic expansion, molecular structure, hyperfine splitting

## Abstract

We report the observation and analysis of the rotational spectrum of a 1:1 cluster between 2-aminopyridine and water (AMW) carried out with supersonic expansion Fourier transform microwave spectroscopy at 4.7–16.5 GHz. Measurements of the 2-aminopyridine monomer (AMP) were also extended up to 333 GHz for the room-temperature rotational spectrum and to resolved hyperfine splitting resulting from the presence of two ^14^N quadrupolar nuclei. Supersonic expansion measurements for both AMP and AMW were also carried out for two synthesized isotopic species with single deuteration on the phenyl ring. Nuclear quadrupole hyperfine structure has also been resolved for AMW and the derived splitting constants were used as an aid in structural analysis. The structure of the AMW cluster was determined from the three sets of available rotational constants and the hydrogen bonding configuration is compared with those for clusters with water of similarly sized single-ring molecules. Experimental results aided by quantum chemistry computations allow the conclusion that the water molecule is unusually strongly bound by two hydrogen bonds, OH**^...^**N and O**^...^**HN, to the NCNH atomic chain of AMP with the potential to replace hydrogen bonds to the identical structural segment in cytosine and adenine in CT and AT nucleic acid base pairs.

## 1. Introduction

**Figure 1:**
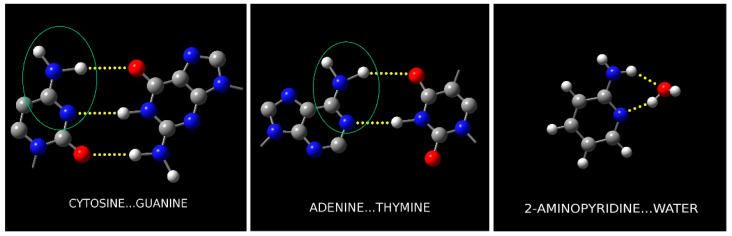
Illustration of the analogies between the hydrogen bonding configuration in the nucleic acid base pairs and AMW. The H_2_NCN atomic segment is present in each left hand molecule, and so is the top NH**^...^**O hydrogen bond. The N**^...^**HN hydrogen bond closing the intermolecular ring in nucleic acid chains is replaced by the N**^...^**HO hydrogen bond in 2-aminopyridine...water.

**Figure 2:**
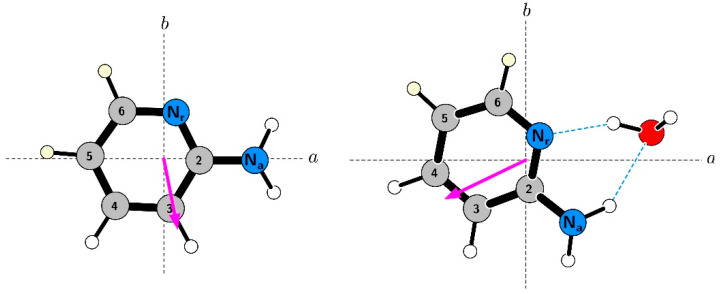
Orientation of AMP and of AMW, and of their electric dipole moments in the principal inertial axes. The dipole moment arrows are drawn from the notional negative to the notional positive charge and it can be seen that the *μ*_b_ component is dominant in AMP, whereas the *μ*_a_ component is dominant in AMW. The orientations of the quadrupole tensors on the two nitrogen atoms, the ring nitrogen N_r_ (with *z* axis close to the ∠CNC bisector), and the amino nitrogen N_a_ (with *z* axis close to the C–NH_2_ bond), are also significantly different in AMP and in AMW. The substitution sites for the singly deuterated isotopologues of AMP, d-5, and d-6, synthesized in this work and used in both AMP and AMW measurements are marked in yellow.

Hydrogen bonding is a fundamental interaction found in many biochemical systems. One area highlighting its importance is the structure of deoxyribonucleic acid (DNA) [1]. Its two helical component chains are connected by hydrogen bonds between the coding nucleic acid residues (see Figure 1), which allows their relatively low-energy separation. 2-aminopyridine (AMP, C_5_H_6_N_2_) is a biomimetic molecule of particular significance since its –NC(NH_2_)– ring segment is identical to those in adenine and cytosine, which is responsible in the latter for two of the three bridging hydrogen bonds in the GC nucleic acid base pair, and for the two bridging bonds in the AT base pair. Formation of the 2-aminopyridine water cluster (AMW) is calculated to involve two hydrogen bonds to the same H_2_N–CNC– molecular segment that is present in both cytosine and in adenine. The first hydrogen bond, N–H**^…^**O, directly mimics the nucleic acid situation. The second intermolecular bond, O–H**^…^**N, subtended by the water moiety in AMW, is also a clear hydrogen bond, similar to that in pyrrole-water [2]. In this way, AMW differs from the previously studied complexes between water and heteroaromatic molecules, such as those with pyrimidine [3], pyrrole [2], or pyrazine [4] in all of which there is only one *bona fide* hydrogen bond, sometimes augmented by a peripheral dispersive interaction (see further below). Hence, the AMW cyclic complex is more successful at mimicking the situation in more complicated biological systems and may offer detailed molecular insight into the solvation of nucleic acid bases. Observation and determination of the properties of AMW was facilitated, in the first step, by a more detailed study of the AMP monomer. The aim was to determine precise rotational constants, centrifugal distortion constants, and especially the nuclear quadrupole splitting constants for the two nitrogen nuclei, all of which can provide useful information on the structure of the AMW cluster. The most direct structural information is provided by the use of several isotopic species of AMW on complex formation. The water molecule and the amino group can be easily deuterated with the use of D_2_O. Unfortunately, both sites are susceptible to deuterium exchange unavoidably producing a complex mixture of isotopic species. On the other hand, deuteration on the aromatic ring is not labile, but it has to be carried out by dedicated chemical synthesis. Presently, two additional ring deuterated isotopic species of AMP have been synthesized (see Figure 2 for positions) and were used in measurements of their monomeric spectra and of spectra of the corresponding isotopic variants of AMW.

## 2. Results

The present studies were carried out with two different spectroscopic techniques. The first was millimeter wave (MMW) spectroscopy at room temperature, which was used to obtain more precise spectroscopic constants of the AMP monomer. The second technique was Fourier Transform Microwave Spectroscopy (FTMW) of supersonic expansion. This complemented the MMW technique since it allowed resolving and measurement of the dual nitrogen nuclear quadrupole hyperfine structure in AMP. This technique was also used for the measurement of the two deuterated species of AMP and for observation and characterization of the AMW cluster and its isotopologues.

### 2.1. MMW Spectroscopy of AMP

There have been two previous investigations of the rotational spectrum of AMP. Kydd and Mills [5] used Stark modulation spectroscopy of the centimeter-wave (CMW) region determining that the molecule displays the effects of a relatively low barrier inversion of the amine group, such that the ground state is split into two sublevels, 0^+^ and 0^−^, with the upper 0^−^ sublevel estimated to lie 125(25) cm^−1^ above the 0^+^ sublevel. They have measured several ^b^R- and ^b^Q-branch transitions at 27.7–37.3 GHz and have also determined the electric dipole moment in the ground 0^+^ sublevel as *μ*_tot_ = 0.88(4) D, with components *μ*_b_ = 0.86(4) D and *μ*_a_ = 0.166(10) D. Ye and Bettens [6] later extended measurements to further *b*-type transitions at 76.1–109.9 GHz, determining quartic centrifugal distortion constants.

**Figure 3:**
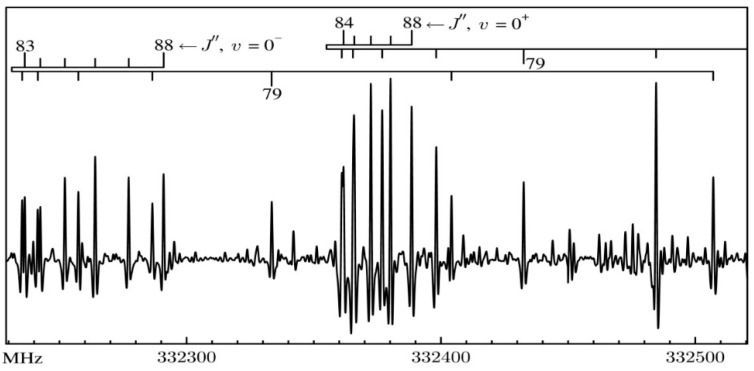
An example of the room temperature MMW-spectrum of AMP, illustrating the characteristic spectral features. Each of the prominent bands marked with overbars consists of R-branch transitions with decreasing value of *J* and increasing values of *K_a_* quantum numbers. The spacing between successive bands of this type depends on the rotational constant *C* and is equal to 2*C* or 3.7 GHz for AMP. The more intense of the two marked bands is due to the lower inversion sublevel 0^+^ of AMP, which is effectively the ground state. The weaker band corresponds to the upper inversion sublevel 0^−^ positioned according to Ref. [5] at 135(25) cm^−1^ above 0^+^.

Presently we extended the measurements of the spectrum of AMP to 258.1–332.8 GHz, that is, to the onset of the submillimeter wave region. The aim has been to enable predictions of the spectrum for possible astrophysical applications and to integrate new data with supersonic expansion measurements (see further below) in order to derive the most precise values of spectroscopic constants for use in structural analysis. At near-submillimeter frequencies and the correspondingly large quantum number values the spectra of near planar molecules are characterized by so-called type-II bands made up of R-branch rotational transitions. Bands of this type also dominate the spectrum of AMP, as illustrated in Figure 3. The onset of such bands in the CMW region was identified by Borchert [7], who initiated the terminology in order to distinguish from type-I bands, in which transitions are for the same value of *J*”. Band evolution towards dense clumps of lines was later described in connection with the chlorobenzene molecule [8], while band properties were generalized in [9,10]. Type-II bands are most prominent in high-*J* rotational spectra of near planar molecules and the distribution of lines in each band is critically dependent on the value of the inertial defect Δ_i_ = *I*_c_ − *I*_a_ − *I*_b_. The differences in the appearance of the 0^+^ and 0^−^ bands in Figure 3 are due to a relatively small change in the inertial defect, with values for the two states being −0.2642 and −0.4390 uÅ^2^, respectively.

Analysis of transitions measured in the MMW spectrum was facilitated by the use of spectroscopic constants from preceding work [5,6] and the evolution of the precision in the determined spectroscopic constants for AMP is illustrated in Table 1.

The present expansion of the upper frequency limit of the spectroscopic data by a factor of 3 not only leads to a corresponding improvement in the precision of the determined rotational constants, but to improvement in the precision of the quartic constants by factors varying from 11 to 23. At the same time it turns out that only one sextic centrifugal constant, *H_K_*, needed to be introduced, and its fitted value is close to *H_K_* = 0.000872 Hz from anharmonic force field computation (at the B3LYP−GD3/6−311+G(2d,p) level). The centrifugal distortion constants for the 0^+^ and 0^−^ sublevels are very close to each other suggesting, together with *H_K_*, that the rotational levels in these vibrational sublevels are sufficiently separated so as not to require additional coupling contributions from the inversion motion. The high frequency spectra also allowed the measurement of some of the weaker ^a^R-branch transitions. The deviations of all fits are consistent with the estimated frequency measurement accuracy of 50 kHz for the employed spectrometers.

### 2.2. Supersonic Expansion FTMW Spectroscopy of AMP and Its Isotopologues

**Figure 4:**
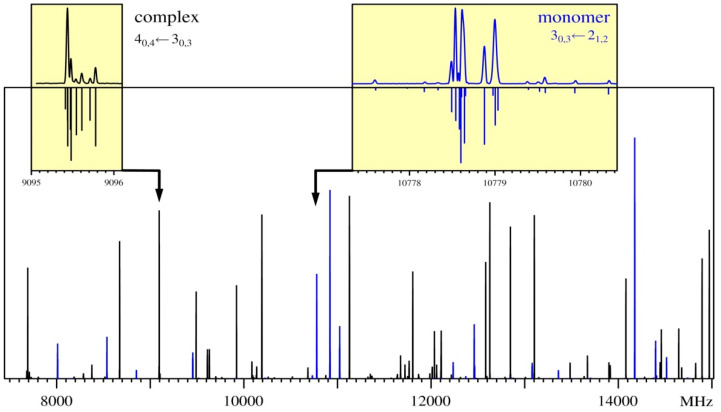
The predicted 1 K rotational spectrum of AMP-d-6 (blue) and AMW-d-6 (black). The two upper boxes provide a comparison of measurement and calculation from the final results of the fits for selected rotational transitions of these isotopic species of AMP and AMW. Each rotational transition carries a dense hyperfine structure due to the presence of the two ^14^N nuclei in the AMP molecule. The inset spectra were measured in the scanning mode of the cavity-FTMW spectrometer.

Cavity FTMW spectroscopy of supersonic expansion allows observation of the sample at an effective rotational temperature of around 1 K so that complicated but compact nuclear quadrupole splitting structures resulting from the presence of two ^14^N nuclei in AMP (and AMW) can be successfully resolved and measured. This is illustrated in Figure 4 for the AMP-d-6 isotopologue.

We have, therefore, used the FTMW method to complement the lowest-*J* measurements of the rotational spectrum of the parent AMP isotopologue and to determine for the first time the nuclear quadrupole coupling constants for its two nitrogen nuclei. All of the available measurements, for hyperfine resolved and hyperfine unresolved transitions have been combined into a single global data set with the results of fit reported in Table 2 (and in more detail in Table S1). There are 83 distinct hyperfine components in the FTMW subset of the global fit, belonging to 16 different rotational transitions, comprising of ^b^R-, ^b^Q-, and ^a^R-branch transitions. The supersonic expansion data subset is fitted to close to the estimated frequency measurement accuracy of 2 kHz for our cavity FTMW spectrometer.

Table 2 and Table S1 also report spectroscopic constants determined for the two synthesized singly deuterated isotopic species of AMP, in their case based only on supersonic expansion measurements. The determined nitrogen coupling constants provide a sensitive double check on the assignment. It can be seen from Figure 2 that both N_a_ and the hydrogen on the C5 site are close to the *a*-axis so that d-5 substitution will not induce a significant rotation of the inertial axes. Hyperfine constants for both nitrogen atoms would, therefore, not be expected to change significantly on d-5 substitution, as is indeed the case. The situation for d-6 substitution is different since this leads to about 2° isotopic rotation, with a visible effect on hyperfine parameters of N_r_ for AMP d-6. In fact, rotation of the N_r_ quadrupole tensor for the parent species by 2° results in (3/2)*χ_aa_* = 0.222 MHz, and (*χ_bb_ − χ_cc_*)/4 = −1.208 MHz, in excellent agreement with the AMP d-6 values in Table 2. The necessary rotations of the quadrupole tensors were performed with programs QDIAG and QPRINC from the PROSPE website [11,12].

### 2.3. Supersonic Expansion FTMW Spectroscopy of AMW and Its Isotopologues

The rotational spectrum of AMW has not, to our knowledge, yet been observed. There is, however, an ion depletion study [13], which reported electronic and vibrational spectra of AMW and identified as the most stable conformation the strongly bound cyclic structure shown in Figure 2. Several other conformers of AMW have also been considered computationally [13], but their binding energies were in all cases lower by 30% or more than for the cyclic structure.

Rotational spectroscopy is able to provide further details concerning the hydrogen bonding configuration, from precisely determined rotational and nuclear quadrupole coupling constants. Supersonic expansion FTMW observation and measurements of the AMW cluster were carried by keeping the AMP sample in a heated stainless steel container upstream of the expansion nozzle and using water premixed in the carrier gas. We were able to observe the transition types anticipated from computations, as implied by the dipole moment orientation in Figure 2, namely stronger ^a^R-branch transitions, and also weaker ^b^R-branch transitions. Measurements of nuclear quadrupole hyperfine components for 23 rotational transitions allowed determination not only of rotational and hyperfine constants but also of all five quartic centrifugal distortion constants for parent AMW, as listed in Table 3 (and in more detail in Table S2). In similarity to FTMW measurements for AMP the fit for AMW is close to the estimated 2 kHz experimental frequency measurement accuracy.

The spectra of the two AMW species incorporating deuterated AMP monomers were measured less extensively, but for a sufficient number of rotational transitions to determine values of the three rotational constants and of the diagonal hyperfine coupling constants. Other spectroscopic parameters were fixed at values for the parent isotopic species. We note that the deviations of fit for the two deuterated species (see Table 3 and Table S2) are significantly poorer than for the parent species. This is attributed to be due to the spin of the deuterium atom which introduces additional frequency contributions and further small splitting, causing many lines to have complex and unresolved structures. Our measurements were made by determining central transition frequencies and fitting the data with the same two-spin model that was used for the parent species. Similar poorer deviations of fit are apparent for the deuterated monomers of AMP (see Table 2), but in all cases the effect on precision of the determined rotational constants is small.

Comparison with computations of the experimental spectroscopic constants for AMP and AMW is made in Table 4 and is unambiguous in confirming the assumed conformation of AMW. We note that for the AMP monomer it is the DFT result that is closer to the experimental (ground state) rotational constants, while for the AMW cluster this is the case for the MP2 computation. The average agreement between the quartic centrifugal distortion constants is better than 3% for AMP and 9% for AMW. This is indicative of the rigidity of both AMP and of the AMW cluster since any large amplitude motions lead to effective contributions that produce significant departures of experimental centrifugal distortion parameters from values calculated on the basis of the harmonic force field.

## 3. Hyperfine Coupling Constants

Consideration of the determined nuclear quadrupole coupling constants for AMP and AMW allows several inferences concerning cluster properties. It is best to start comparisons, as made in Table 5, from the *χ_cc_* constant, since this parameter is along the axis perpendicular to the AMP or AMW plane. It should be invariant to uncertainties concerning the rotation of AMP in this plane, which may affect values of *χ_aa_* and *χ_bb_*. Changes in *χ_cc_* between AMP and AMW will, therefore, directly reflect the effects of complexation.

Comparison of the experimental values of *χ_cc_* for the amino group nitrogen N_a_ in AMP and in AMW reveals that those are quite similar, with only a 2% change (−4.203 and −4.114 MHz, respectively). Furthermore, rotation of the experimental AMP hyperfine tensor for N_a_, as tabulated in the rightmost column of Table 5, is in reasonable, though worse, correspondence with the values for AMW. On the other hand, complexation change in experimentally determined *χ_cc_* of the ring nitrogen N_r_ is much greater, at nearly 20% (from 2.343 MHz in AMP to 1.882 MHz in AMW). This indicates much greater field gradient modification by hydrogen bonding to this nitrogen, than is the case for hydrogen bonding to the N_a_H_2_ group.

Relatively routine level quantum chemistry computations, such as those used in this work, provide satisfactory reproduction of experimental nuclear quadrupole coupling constants but usually only once scaling is used [14]. We find, for the B3LYP−GD3/6−311+G(2d,p) calculations used here, that the scaling factor of 0.941 reproduces *χ_cc_* for both nitrogen nuclei in AMP almost exactly. The computed values used in various comparisons made in Table 5 all result from scaling with this factor. The comparison of experiment and computation for both nitrogen nuclei in AMP and AMW is reasonable. The results for N_r_ in AMW are now much better than when obtained by rotation of the molecular tensor from AMP. This type of comparison has often been used in the identification of conformations in nitrogen-containing molecules of biological relevance, such as cytosine [15] or ephedrine [16].

Nuclear quadrupole coupling constants can also carry angular information. This is the case when some off-diagonal constants can be determined, such as *χ_ab_* of the N_r_ nucleus in AMW. It then becomes possible to diagonalize the hyperfine tensor determined in the principal inertial axes *a*, *b*, *c* to its principal quadrupolar axes *x*, *y*, *z*. For a nitrogen atom in the *ab* inertial plane, the diagonalization operation corresponds to axes rotation in this plane by angle *θ_za_*, determined on diagonalization. The correspondence between this angle and the structure of the molecule has been investigated in detail for halogen nuclei terminal to a chemical bond [17]. It was found that there is a small difference between *θ_za_* and the bond axis, but that such difference is easily calculable. This has been used in structural evaluations in several other molecules [18,19,20], as well as in the (HCl)_2_H_2_O cluster [21]. Presently we note that the structural angle *θ*_str_ = 17.5° between the ∠(CNC) bisector and the *a*-principal inertial axis evaluated from the quadrupole information for N_r_ compares well with the angle *θ*_str_ = 19.2° from the structure of AMW evaluated in the next section.

## 4. Structure of AMW

**Figure 5:**
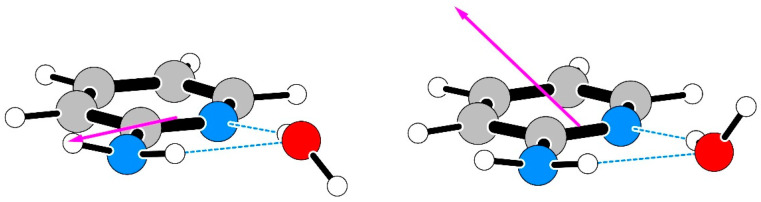
The calculated geometries of the U/D conformer of AMW (**left**) and of the U/U conformer (**right**) and the orientation of their electric dipole moments.

Quantum chemistry calculations reveal two possible geometries for AMW as depicted in Figure 5. These are U/D and U/U such that in both configurations the amino hydrogens are Up and the non-bonded water hydrogen can be either Down or Up. These two AMW geometries are very close in energy, although U/D is always the more stable, with the energy difference ranging from 30 to 100 cm^−1^, depending on the details of the computation (such as whether BSSE and ZPE effects have been accounted for or not, or whether simple enthalpies or Gibbs free energies are compared). The most direct indicator, however, seems to be the electric dipole moment. In the U/U conformer, there is expected to be a significant *μ*_c_ component, unlike the practically zero value in the U/D conformer. No *c*-type rotational transitions have been observed so that the most probable structure is U/D.

There are many different ways of evaluating cluster geometries. The simplest one, if a sufficient number of single isotopic substitutions is available, is to use the Kraitchman substitution method [22]. This is, unfortunately, very susceptible to the neglect of intermolecular vibrational motions and, in any case, we do not have suitable substituted species in hand. The alternative is to use one of several least-squares structure determination methods, which allow the evaluation of partial geometries when augmented by quantum chemistry computations. These include effective ground state (*r*_0_) geometries, mass scaled (*r*_m_) geometries [23], or the most sophisticated semi-experimental (*r*_e_^SE^) geometries [24,25]. In the present case, only determination of the *r*_0_ geometry is practicable, but this has been found to be a good choice for determining heavy atom hydrogen bond distances in the more strongly bound clusters, such as those involving various numbers of water molecules from the hexamer upwards [26,27,28]. In the present determination, we use the ground state monomer geometry for water [29] and the B3LYP−GD3/6−311+G(2d,p) geometry of AMP scaled to reproduce ground state rotational constants. The scaling was carried out with program CORSCL from the PROSPE website, which allows separate scaling along each principal axis by matching observed and scaled planar moments. Several simplifying assumptions concerning the relative orientation of H_2_O and AMP were made, since experimental results are not sensitive to such details. Thus the nonlinearity in the O**^…^**HN_r_ hydrogen bond was assumed from the MP2/aug-cc-pVDZ computation, and this bond was also assumed to lie in the plane of AMP. As a result, fitting of the relative orientation of the two partner molecules was reduced from six parameters for a general situation to only three. This gave rise to a very well convergent fit, with key results reported in Table 6.

The uncertainties on the three parameters of the fit are seen to be very small, although those are only meaningful in the statistical sense. Realistic uncertainties may up to an order of magnitude greater in view of the various approximations concerning monomer geometries and their relative orientation. Even when this is taken into account the precision of the experimental result is very satisfactory and the comparison with computation is seen to be very good.

## 5. Discussion

**Figure 6:**
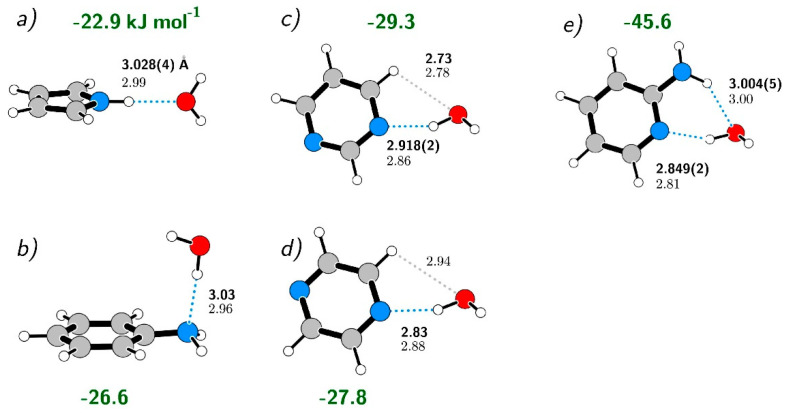
Comparison of conformations, hydrogen bond heavy atom distances, and of calculated Basis Set Superposition Error (BSSE) corrected complexation energies (in green) for several related clusters between single ring aromatic molecules and a water molecule bound by NH**^…^**O and/or N**^…^**HO hydrogen bonds. The structures are drawn on the basis of published data for (**a**) Pyrrole**^…^**H_2_O [2]. (**b**) Aniline**^…^**H_2_O [31]. (**c**) Pyrimidine**^…^**H_2_O [3]. (**d**) Pyrazine**^…^**H_2_O [4]. (**e**) AMW, this work. Interatomic distances (where available) consist of the reported experimental values in bold font, with computed values directly below, in normal font. All computed values have been evaluated presently at the B3LYP−GD3/6311+G(2d,p) level. Dotted blue lines mark hydrogen bonds, while grey dotted lines mark secondary intermolecular bonds in which atomic separation is close to the sum of van der Waals radii.

The AMW cluster studied presently is an interesting, and potentially very relevant, addition to our knowledge concerning clusters between water and monocyclic aromatic molecules with a nitrogen atom either in the ring or in a substituent. A useful comparison with pyrrole**^…^**H_2_O [2], aniline**^…^**H_2_O [31], pyrimidine**^…^**H_2_O [3] and pyrazine**^…^**H_2_O [4] illustrating some systematic trends is presented in Figure 6. We concentrate on heavy atom separations in the hydrogen bond since details concerning the hydrogen position in such a bond are usually not clear from rotational spectroscopy, even with a complete set of deuterium substitutions [27]. Heavy atom separation is inversely proportional to hydrogen bond strength and the latter is also listed for the clusters in Figure 6. The level of computation used here in combination with the BSSE reduction with the counterpoise method [32] was calibrated using the much-studied water dimer. Presently evaluated dimerization energy of −23.4 kJ/mol, can be compared with more specialized MP2 and RI-MP2 determinations at the complete basis set limit of –20.8 kJ/mol [33] and −21.1 kJ/mol [34]. This suggests that we risk only a relatively small overestimate in relation to more sophisticated computations.

The clusters on the left of Figure 6, pyrrole**^…^**H_2_O and aniline**^…^**H_2_O, have the best-defined single hydrogen bonds to the water molecule. The two middle clusters involving pyrazine and pyrimidine, are somewhat more strongly bound due to additional peripheral interaction at van der Waals distance between water oxygen and hydrogen on the aromatic ring. The presently studied AMW cluster is much more strongly bound, with binding energy not far from the sum of those for the two leftmost clusters. The N**^…^**HO distances are consequently steadily decreasing in the sequence of clusters of water with aniline, through pyrimidine, and finally AMP. The trend is the same for experiment and computation. The NH**^...^**O distances in pyrrole**^…^**H_2_O and in AMW are very similar, again both in experiment and computation. The only exception to the trends seems to be pyrazine**^…^**H_2_O, with a somewhat short experimental N**^…^**O distance, and that may merit reinvestigations. Computations suggest that this is less strongly bound than pyrimidine**^…^**H_2_O and that the N**^…^**O distance in pyrazine**^…^**H_2_O may be longer. Investigation of all clusters shown in Figure 6 was particularly favorable experimentally, since near ambient conditions were sufficient in order to obtain gas-phase monomer populations suitable for supersonic expansion. Studies of larger clusters, more closely approximating biological systems, are hindered by very low volatility of many candidate molecules, so that more powerful laser ablation techniques as used, for example, for cytosine [15], are necessary.

In summary, the binding of water to AMP is particularly strong, taking place in a cyclic way via two *bona fide* hydrogen bonds. The H_2_N–CNC structural segment in AMP is identical to those in cytosine and adenine, which also subtend two hydrogen bonds to partner nucleic acid bases in the genetic coding scenario. It is thus very likely that the single hydration step from AMP to AMW may be in a significant analogy to processes associated with DNA replication.

## 6. Materials and Methods

The parent isotopic species of 2-aminopyridine was a commercially available sample used without further purification. The two deuterated species were synthesized chemically as follows. In a round bottomed flask, 5- or 6-bromo-2-aminopyridine (3.6 g, 2.2 mmol), a solution of deuterated water (35 mL) and dideuterosulfuric acid (98%, 4 mL), and Zn in powder (7 g) were introduced under nitrogen. The mixture was stirred under reflux for 2 h and then cooled and filtered. The solid residue was washed three times with water (20 mL). The water was removed in vacuo. A 40 % NaOH aqueous solution (40 mL) was then added and the organic compounds were extracted with dichloromethane (3 × 50 mL). The organic phase was dried on MgSO_4_ and the solvent was removed in a vacuum. A pure 5- or 6-deutero-2-aminopyridine respectively was thus obtained in a 65% yield (1.22 g, 1.44 mmol).

The MMW measurements were made at room temperature with the broadband, Backward Wave Oscillator based spectrometer in Warsaw [35], updated to use more convenient detectors and harmonic mixers as described in [36]. In this case, the vapor pressure available above the room-temperature sample of AMP was sufficient.

Supersonic expansion FTMW measurements were carried out with the cavity FTMW spectrometer [37] in Warsaw. This is a coaxial version of the pioneering Balle, Flygare design [38], and was modified for single frequency signal downconversion [39]. We aimed to obtain a 1% mixture of AMP in carrier gas at near 1 atm pressure, and this required heating the sample of AMP held in a stainless steel sample tube to ca 60–80 °C. The carrier gas was either pure Ar or 1:2 Ar:He mixture, as that was found to produce significant signal improvement for monomer spectra [40]. Water for AMW spectra was usually premixed with a carrier gas, but it was also found that water absorbed by AMP was sufficient for good cluster signals. The analysis was carried out with the help of the graphical AABS package for synchronously displaying recorded spectra and predictions [41,42], which is available on the PROSPE website [11,12]. Fits and predictions were carried out either with ASFIT/ASROT package [43], also available on the PROSPE website or with the SPFIT/SPCAT package of H.M. Pickett [44,45]. Least-squares structural fits were performed with the program STRFIT [30].

Quantum mechanical computations were carried out with Gaussian [46], CFOUR [47], or Firefly [48] packages. Dissociation energies were evaluated by correcting for BSSE using the counterpoise method [32].

## Figures and Tables

**Table 1:** Spectroscopic constants determined for the 2-aminopyridine molecule from room temperature rotational spectroscopy.

	0^+^	0^+^	0^+^	0^−^
Parameter ^a^	Ref. [5]	Ref. [6]	This Work	This Work
*A*/MHz	5780.34	5780.3720(10) ^b^	5780.37487(28)	5778.56502(30)
*B*/MHz	2733.57	2733.50354(35)	2733.50446(12)	2730.18938(14)
*C*/MHz	1857.66	1857.67392(40)	1857.675172(98)	1857.14913(12)
				
*Δ_J_*/kHz		0.13653(27)	0.137392(13)	0.137264(16)
*Δ_JK_*/kHz		0.2029(11)	0.202586(48)	0.203231(70)
*Δ_K_*/kHz		0.9312(44)	0.93717(23)	0.93003(27)
*δ_J_*/kHz		0.045827(74)	0.0458083(43)	0.0456266(56)
*δ_K_*/kHz		0.36733(99)	0.365545(88)	0.364984(95)
				
*H_K_*/Hz			0.000643(56)	0.000571(65)
				
*N*_trans_	9	148	261	234
*σ*_fit_/kHz		46.79	43.97	48.43

^a^*A*, *B* and *C* are rotational constants. Δ*_J_* to *H_K_* are centrifugal distortion constants, *N*_trans_ is the number of distinct frequency measured lines in the fit, and *σ*_fit_ is the deviation of the fit. ^b^ Quantities in brackets are standard errors in units of the last digit of the value of the constant.

**Table 2:** Spectroscopic constants determined for the AMP molecule and its isotopic species deuterated on the phenyl ring.

Parameter	Parent AMP ^a^	AMP-d-5 ^b^	AMP-d-6 ^b^
*A*/MHz	5780.374597(100)	5778.43386(44)	5485.85599(45)
*B*/MHz	2733.504446(67)	2613.82591(45)	2700.45410(55)
*C*/MHz	1857.675169(52)	1801.46262(16)	1811.36749(17)
			
*Δ_J_*/kHz	0.137393(10)	c	c
*Δ_JK_*/kHz	0.202569(47)	c	c
*Δ_K_*/kHz	0.93701(16)	c	c
*δ_J_*/kHz	0.0458083(39)	c	c
*δ_K_*/kHz	0.365563(90)	c	c
			
*H_K_*/Hz	0.000605(43)		
			
N_a_: (3/2)*χ_aa_* ^d^/MHz	3.5854(17)	3.6005(54)	3.5732(79)
N_a_: (*χ_bb_ − χ_cc_*)/4 /MHz	1.50413(70)	1.5056(20)	1.5021(27)
N_a_: *χ_cc_*/MHz	−4.2034(15)	−4.2114(44)	−4.1953(60)
			
N_r_: (3/2)*χ_aa_*/MHz	−0.0789(26)	−0.048(11)	0.226(12)
N_r_: (*χ_bb_ − χ_cc_*)/4 /MHz	−1.15823(85)	−1.1695(25)	−1.2171(35)
N_r_: *χ_cc_*/MHz	2.3428(19)	2.3550(62)	2.3589(81)
			
*N*_trans_	344	43, 8 ^e^	41, 8 ^e^
*σ*_fit_ /kHz	42.89 + 2.23 ^f^	3.91	4.35

^a^ Global fit to FTMW, MMW and CMW data using A-reduced Watson’s asymmetric rotor Hamiltonian in representation I^r^ combined with nuclear quadrupole coupling terms in the **I**_tot_ = **I**(N_a_) + **I**(N_r_) coupling scheme. ^b^ Fit based only on FTMW measurements. ^c^ Value of the parameter assumed to be the same as for the parent isotopic species. ^d^ The hyperfine constants (3/2)*χ_aa_* and (*χ_bb_* − *χ_cc_*)/4 are direct parameters of fit, while *χ_cc_* is derived from the parameters of fit using the Laplace’s relation *χ_aa_* + *χ_bb_* + *χ_cc_* = 0. ^e^ The number of distinct fitted frequencies, and the number of different measured rotational transitions, respectively. ^f^ Deviations of fit to the MMW + CMW subset, and to the FTMW subset of data, respectively.

**Table 3:** Spectroscopic constants determined for the AMW cluster and its isotopologues.

Parameter	Parent AMW ^a^	AMW-d-5 ^a^	AMW-d-6 ^a^
*A*/MHz	3722.8201(14)	3640.3373(12)	3558.6210(28)
*B*/MHz	1384.23544(25)	1354.60499(75)	1383.47362(34)
*C*/MHz	1011.15836(18)	989.28824(67)	998.26318(37)
			
*Δ_J_*/kHz	0.2058(20)	0.2533(94)	0.2279(57)
*Δ_JK_*/kHz	0.419(16)	b	b
*Δ_K_*/kHz	2.21(29)	b	b
*δ_J_*/kHz	0.0588(14)	b	b
*δ_K_*/kHz	0.612(47)	b	b
			
N_a_: (3/2)*χ_aa_*/MHz	3.0091(33)	3.113(68)	2.975(35)
N_a_: (*χ_bb_ − χ_cc_*)/4 /MHz	1.55556(100)	1.542(15)	1.5513(89)
N_a_: *χ_cc_*/MHz	−4.1142(23)	−4.122(38)	−4.094(24)
			
N_r_: (3/2)*χ_aa_*/MHz	−4.8756(28)	−4.821(34)	−4.816(22)
N_r_: (*χ_bb_ − χ_cc_*)/4 /MHz	−0.1284(11)	−0.1455(89)	−0.1256(87)
N_r_: *χ_ab_*/MHz	1.43(19)	b	b
N_r_: *χ_cc_*/MHz	1.8818(22)	1.894(28)	1.857(22)
			
*N*_trans_^c^	97, 23	34, 10	38, 11
*σ*_fit_ /kHz	1.96	5.38	3.86

^a^ Fit to only the FTMW measurements. ^b^ Value of the parameter assumed to be the same as for the parent isotopic species. ^c^ The number of distinct fitted frequencies, and the number of different measured rotational transitions, respectively.

**Table 4:** Comparison of experimental and computed spectroscopic constants for AMP and AMW.

		AMP			AMW	
	Exp.	Calc. ^a^	Calc. ^b^	Exp.	Calc. ^a^	Calc. ^b^
*A*^c^/MHz	5780.374597(100)	5814.9	5689.1	3722.8201(14)	3701.4	3652.2
*B*/MHz	2733.504446(67)	2736.4	2689.2	1384.23544(25)	1410.8	1384.5
*C*/MHz	1857.675169(52)	1863.2	1829.3	1011.15836(18)	1027.0	1010.9
						
*Δ_J_*/kHz	0.137393(10)	0.1335	0.1309	0.2058(20)	0.1734	0.1870
*Δ_JK_*/kHz	0.202569(47)	0.2038	0.2029	0.419(16)	0.4308	0.3419
*Δ_K_*/kHz	0.93701(16)	0.9198	0.8659	2.21(29)	2.145	2.173
*δ_J_*/kHz	0.0458083(39)	0.04440	0.04376	0.0588(14)	0.04755	0.04985
*δ_K_*/kHz	0.365563(90)	0.3573	0.3528	0.612(47)	0.5924	0.5861

^a^ Computed from the B3LYP−GD3/6−311+G(2d,p) harmonic force field. ^b^ Computed from the MP2/aug-cc-pVDZ harmonic force field. ^c^ Experimental parameters are for the ground state (0^+^ sublevel for AMP), while the computed values are for equilibrium.

**Table 5:** Comparison of experimental and calculated nuclear quadrupole coupling constants for the two nitrogen nuclei in AMP and AMW.

Parameter	Parent AMP		Parent AMW		
	Exp.	Calc. ^a^	Exp.	Calc. ^a^	Calc.
N_a_: *χ_aa_* /MHz	2.3903(11)	2.300	2.0061(21)	1.991	2.171 ^b^
N_a_: *χ_bb_* /MHz	1.8132(15)	1.903	2.1081(23)	2.087	2.039 ^b^
N_a_: *χ_cc_* /MHz	−4.2034(15)	−4.203	−4.1142(23)	−4.078	−4.203 ^b^
					
N_r_: *χ_aa_* /MHz	−0.0526(17)	−0.035	−3.2504(19)	−3.249	−3.68 ^c^
N_r_: *χ_bb_* /MHz	−2.2903(19)	−2.309	1.3684(24)	1.481	1.34 ^c^
N_r_: *χ_cc_* /MHz	2.3428(19)	2.343	1.8820(24)	1.769	2.34 ^c^
N_r_: *χ_ab_* /MHz	2.92(15) ^d^	2.92	1.43(19)	1.715	0.70 ^c^
					
N_r_: *χ_zz_* /MHz	−4.30(14)	−4.310	−3.66(10)	−3.829	−4.30
N_r_: *θ_za_* ^e^/deg	55.5(5)	55.6	15.9(17)	18.3	18.3
N_r_: *θ*_str_ ^f/^deg			17.5 ^g^	19.9	

^a^ From B3LYP+GD3/6−311+G(2d,p) computation scaled by the factor 0.941 determined from AMP. ^b^ Experimental N_a_ values from AMP rotated by the angle 38.1° between the CN_a_ axis and the *a*-principal inertial axis in the AMW structure fitted below. ^c^ Experimental N_r_ values from AMP rotated by the angle 19.2° between the CN_a_C bisector and the *a*-principal inertial axis in the AMW structure fitted below. ^d^ The calculated scaled value has been assumed and assigned 5% uncertainty. ^e^ *θ_za_* is the angle between the quadrupole tensor in principal inertial axes and in principal quadrupolar axes. ^f^ *θ*_str_ is the angle between the ∠(CNC) bisector and the *a*-principal inertial axis. ^g^ The experimental *θ_za_* value corrected by the difference (*θ*_str_ − *θ_za_*) from computation.

**Table 6:** The results of fitting the geometry of AMW.

	AMW		AMW d-5		AMW d-6	
	Exp.	o.-c.	Exp.	o.-c.	Exp.	o.-c.
*Ia*/uÅ^2^	135.7523	0.0225	138.8246	−0.0317	142.0154	0.0138
*Ib*/uÅ^2^	365.0978	0.0291	373.0822	0.0299	365.2971	−0.0019
*Ic*/uÅ^2^	499.8001	0.0059	510.8523	−0.0054	506.2585	0.0125
			fitted ^a^		calc. ^b^	
	*σ*_fit_^c^ = 0.0247 uÅ^2^		*d*(O^…^N_r_) = 2.8489(2) Å		2.8272	
			A(O^…^N_r_–C2) = 102.71(2)°		101.75	
			D(HOH^…^N_r_) = 140.9(8)°		134.3	

^a^ Results from a three parameter *r*_0_ fit made with STRFIT [30] to the nine experimental moments of inertia. ^b^ Computed at the MP2/aug-cc-pVDZ level. ^c^ Deviation of the structural fit to moments of inertia, corresponding to average deviation of fit of 0.45 MHz to rotational constants.

## Data Availability

Most of the relevant data are contained within the article or supplementary material. In addition, primary input and output files for the performed fits are available at http://info.ifpan.edu.pl/~kisiel/data.htm (accessed on 20 October 2021).

## Supplementary Materials

The following are available online, Table S1: collected results of spectroscopic fits for AMP; Table S2: collected results of spectroscopic fits for AMW.

## Abbreviations

The following abbreviations are used in this manuscript:

AMP	2-aminopyridine
AMW	2-aminopyridine…water cluster
FTMW	Fourier Transform MicroWave spectroscopy of supersonic expansion
MMW	Millimeter-wave
CMW	Centimeter-wave
